# A journey on plate tectonics sheds light on European crayfish phylogeography

**DOI:** 10.1002/ece3.4888

**Published:** 2019-01-19

**Authors:** Lucian Pârvulescu, Jorge L. Pérez‐Moreno, Cristian Panaiotu, Lucian Drăguț, Anne Schrimpf, Ioana‐Diana Popovici, Claudia Zaharia, András Weiperth, Blanka Gál, Christoph D. Schubart, Heather Bracken‐Grissom

**Affiliations:** ^1^ Department of Biology‐Chemistry, Faculty of Chemistry, Biology, Geography West University of Timisoara Timisoara Romania; ^2^ Department of Biology Florida International University – Biscayne Bay Campus North Miami Florida; ^3^ Paleomagnetic Laboratory, Faculty of Physics University of Bucharest Magurele Romania; ^4^ Department of Geography, Faculty of Chemistry, Biology, Geography West University of Timisoara Timisoara Romania; ^5^ Institute for Environmental Sciences University Koblenz‐Landau Landau Germany; ^6^ Department of Mathematics, Faculty of Mathematics and Computer Science West University of Timisoara Timisoara Romania; ^7^ MTA Centre for Ecological Research, Danube Research Institute Budapest Hungary; ^8^ Doctoral School of Environmental Sciences Eötvös Loránd University Budapest Hungary; ^9^ Department of Zoology and Evolutionary Biology University of Regensburg Regensburg Germany

**Keywords:** Apuseni Mountains, biogeographical pattern, divergence time estimates, endemic lineages, freshwater species distribution, molecular clock, Tisza–Dacia mega‐unit

## Abstract

Crayfish can be used as model organisms in phylogeographic and divergence time studies if reliable calibrations are available. This study presents a comprehensive investigation into the phylogeography of the European stone crayfish (*Austropotamobius torrentium*) and includes samples from previously unstudied sites. Two mitochondrial markers were used to reveal evolutionary relationships among haplogroups throughout the species’ distributional range and to estimate the divergence time by employing both substitution rates and geological calibration methods. Our haplotype network reconstruction and phylogenetic analyses revealed the existence of a previously unknown haplogroup distributed in Romania's Apuseni Mountains. This haplogroup is closely related to others that are endemic in the Dinarides, despite their vast geographical separation (~600 km). The separation is best explained by the well‐dated tectonic displacement of the Tisza–Dacia microplate, which started in the Miocene (~16 Ma) and possibly carried part of the *A. torrentium* population to the current location of the Apuseni Mountains. This population may thus have been isolated from the Dinarides for a period of ca. 11 m.y. by marine and lacustrine phases of the Pannonian Basin. The inclusion of this geological event as a calibration point in divergence time analyses challenges currently accepted crayfish evolutionary time frames for the region, constraining the evolution of this area's crayfish to a much earlier date. We discuss why molecular clock calibrations previously employed to date European crayfish species divergences should therefore be reconsidered.

## 1 INTRODUCTION

Tectonics, mountains, and climate are decisive drivers that have shaped the evolution of the earth's terrestrial and freshwater biodiversity and the distribution of different species (Antonelli, 2017). Investigations into the ecological, historical, and evolutionary features of species are of utmost importance when seeking to explain their current distributions (Caplat et al., 2016; Ficetola, Mazel, & Thuiller, 2017; Rodrigues, Olalla‐Tárraga, Iverson, Akre, & Diniz‐Filho, 2017). The distribution of freshwater species is continentally restricted and dependent on freshwater hydrological connections. As such, these distributions are sensitive to perturbations of the surrounding environment (le Roux, Virtanen, & Luoto, 2013; Slaton, 2015) and reflect both historical and recent geological changes (Elith & Leathwick, 2009; Tingley & Beissinger, 2009). Crayfish are ideal biogeographical indicators, since they display limited migration behavior beyond their watershed (Bubb, Thom, & Lucas, 2006) and lack a planktonic larval phase that could be spread by vectors. Crayfish are part of a monophyletic clade that includes the families Astacidae, Cambaridae, and Parastacidae within the decapod infraorder Astacidea, which also includes marine lobsters. Their diversification began ~261–268 Ma and was later facilitated by the breakup of Pangaea (Bracken‐Grissom et al., 2014; Crandall & Buhay, 2008; Crandall, Harris, & Fetzner, 2000; Toon et al., 2010). Phylogeographic reconstructions for crayfish and other freshwater species must consider both their evolutionary origins and the historical development of freshwater habitats (Crandall et al., 2000; Gyllensten, 1985; Tsang et al., 2014).

The Alpine–Carpathian–Dinaric (ACD) orogenic system has been found to have shaped the biogeography of many freshwater species (including crayfish) distributed within central and southeastern Europe (Bálint, Barnard, Schmitt, Ujvárosi, & Popescu, 2008; Copilaş‐Ciocianu & Petrusek, 2015; Jelić et al., 2016; Klobučar et al., 2013; Mráz & Ronikier, 2016; Trontelj, Machino, & Sket, 2005). Among these species, is the stone crayfish *Austropotamobius torrentium* (Schrank, 1803), probably one of the first crayfish lineages to establish in Europe (Crandall et al., 2000). This crayfish has an interesting, while not fully understood, phylogeny. Previous phylogeographic reconstructions have proposed the north‐central Dinarides (NCD) Mountain region as the center of *A. torrentium* radiation, since this region contains the highest genetic diversity for the species (Trontelj et al., 2005), specifically five haplogroups according to Klobučar et al. (2013). Expansion followed to the Balkan region and then spread throughout the Danube Basin (Klobučar et al., 2013). The species is currently present in central and western Europe, from the Balkan Peninsula, through Romania and Hungary to the Czech Republic, Austria, Germany, and across to Luxembourg and eastern France (see distribution map in Kouba, Petrusek, & Kozák, 2014). Some populations in western Europe are suspected to exist as a result of translocation (Fratini et al., 2005; Petrusek et al., 2017; Trontelj et al., 2005).

To date, intraspecific phylogenies of *A. torrentium* have been reconstructed without the inclusion of individuals from the northeastern part of the species' range, the Apuseni Mountain region (APU) in northwestern Romania. Inclusion of these individuals may be essential for estimates of the phylogeography and divergence times, because they are geographically isolated from other populations in the Pannonian Basin (Pârvulescu, Zaharia, Satmari, & Drăguț, 2013) and the geographical unit including the Apuseni Mountains was separated from the Dinarides as a result of tectonic displacement ~16 Ma (see Balázs, Mațenco, Magyar, Horváth, & Cloetingh, 2016; Kováč et al., 2018). Previous studies have calibrated the molecular clock based on geological events, however with less reliable calibration times. For instance, the divergence between species of *Austropotamobius* was timed based on the uplift of the Dinarides (Jelić et al., 2016; Klobučar et al., 2013; Trontelj et al., 2005). However, this process lasted for ~25 Ma (Schmid et al., 2008), an interval that cannot offer a specific anchor for calibration and consequently yields imprecise estimations. Therefore, the incorporation of the isolated APU population, which can be accurately tied into the well‐documented results of tectonic displacement, and the formation of a marine water flooded rift (Balázs et al., 2016; Magyar et al., 2013), has the potential to advance the knowledge toward a full understanding of the evolutionary history of the *A. torrentium*.

This study reconstructs the phylogeny of *A. torrentium* across its distributional range by capitalizing on the opportunity of adding an important missing piece of the puzzle, namely the isolated APU population that can be linked to a specific tectonic event. Specifically, the main questions addressed herein are (a) how is the newly discovered APU population related to the other known populations, and (b) how does incorporation of the APU population, in light of the tectonic history of the region, affects the divergence time estimates of all *A. torrentium *populations?

## 2 MATERIALS AND METHODS

### 2.1 Specimen sampling, DNA extraction, amplification, and sequencing

**Figure 1:**
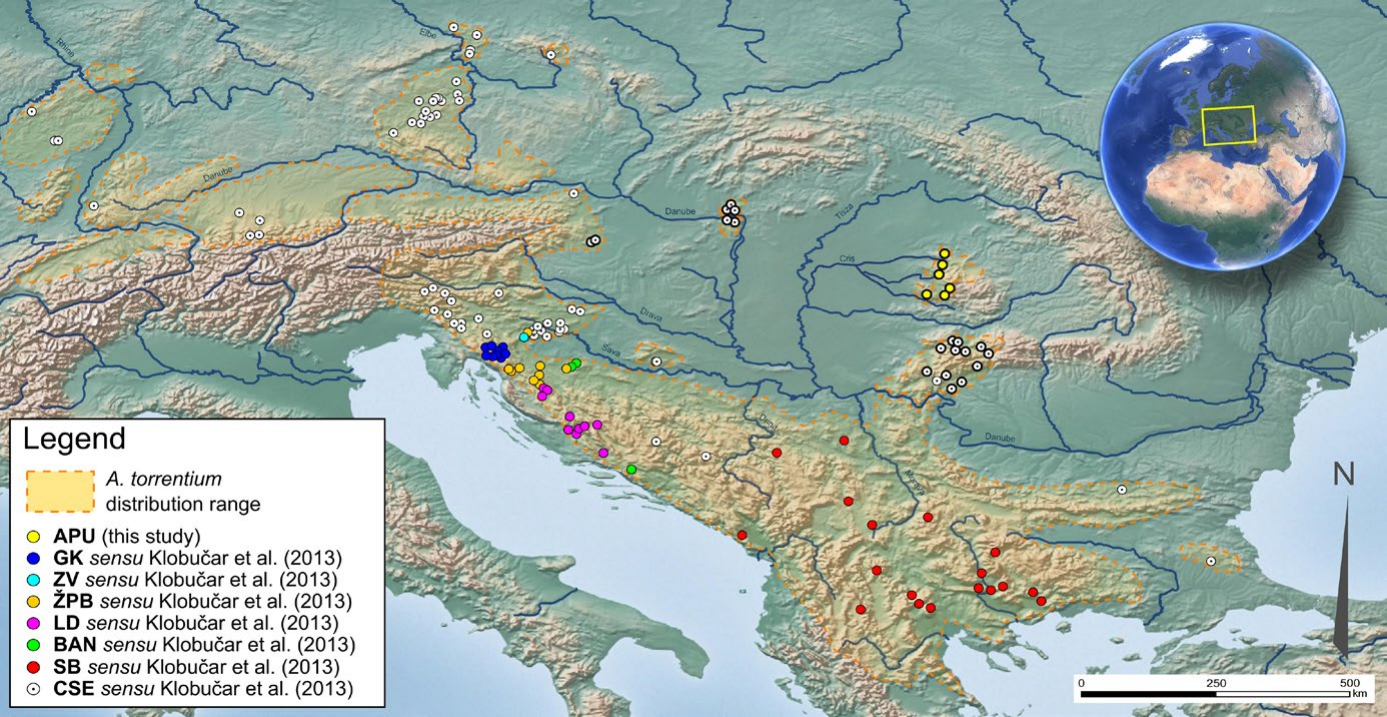
Geographical distribution of *Austropotamobius torrentium* phylogroups in Europe. Bold circles indicate the new sampling sites selected for this study as they cover the entire species range

Tissue for molecular investigations was collected from crayfish captured by hand in the field. A segment of the last pereiopod was cut and preserved in 96% ethanol for later DNA extraction. The sampling sites were chosen in a way to complement previous studies on the *A. torrentium* phylogeography (Figure 1), which failed to include samples from the northeastern limit of the species range (north and east of the Pannonian Plain), referring to the most recent species distribution map of Kouba et al. (2014). In addition to these new samples, available sequences corresponding to the cytochrome c oxidase I (COI) and 16S rRNA mitochondrial genes were downloaded from NCBI's GenBank, with sequences of European astacids (*Astacus astacus, A. leptodactylus; Austropotamobius pallipes, *and *A. italicus*) as outgroups (see Supporting information Table S1).

Genomic DNA was extracted from the crayfish muscle tissue with the Qiagen DNeasy Blood & Tissue Kit (Qiagen, Hilden, Germany) according to the manufacturer protocol, and DNA was stored at −20°C until running the polymerase chain reaction (PCR). Two mitochondrial (mtDNA) gene fragments were amplified: the COI protein‐coding gene with the primer pair LCO‐1490/HCO‐2198 (Folmer, Black, Hoeh, Lutz, & Vrijenhoek, 1994) and the 16S rRNA gene with the primer pair 16Sar/16Sbr (Simon et al., 1994). Those DNA markers were chosen because they are compatible with already existing sequences for *A. torrentium* in GenBank (i.e., Trontelj et al., 2005; Schubart & Huber, 2006; Klobučar et al., 2013; Petrusek et al., 2017). PCR was performed using Qiagen HotStarTaq Master Mix Kit (Qiagen, Germany). Reactions were prepared in a total volume of 50 µl containing: 2.5 U HotStarTaq DNA Polymerase, 1.5 mM MgCl_2_, 200 µM each dNTP, 0.2 µM of each primer, and 20 ng of DNA template. For PCR runs, the following cycling conditions were used: initial denaturation at 95°C for 15 min; 35 cycles of 45 s at 94°C for denaturation, 45 s at 55°C (16S rRNA) or 47°C (COI) for annealing, 60 s at 72°C for extension, and 10 min at 72°C for final extension. Geneious (version 11; Kearse et al., 2012) was used for quality filtering, trimming, and primer removal of trace files, whereby any sequences with low quality and/or ambiguities were excluded from the analyses. To check for pseudogenes, we followed suggestions by Song, Buhay, Whiting, and Crandall (2008), which included translating protein‐coding sequences (COI) to check for indels and stop codons, comparing sequences to closely related species and building individual gene trees to ensure similar topologies. MAFFT (version 7.187; Katoh, Kuma, Toh, & Miyata, 2005) was employed for aligning sequences, which resulted in final alignments of 467 base pairs (bp) for 16S rDNA and 593 bp for COI mtDNA. Additional information and accession numbers for the final data set, including *A. torrentium* mtDNA sequences from GenBank, can be found in Supporting information Table S1.

### 2.2 Haplotype networks

Haplotype networks were constructed for individual and concatenated loci with PopArt 1.7 (Leigh & Bryant, 2015) using TCS (Templeton–Crandall–Sing) networks (Templeton, Crandall, & Sing, 1992). TCS was chosen for its intuitive visualization and its effectiveness at recovering accurate population‐scale phylogeographic patterns (Gerber & Templeton, 1996; Turner, Trexler, Harris, & Haynes, 2000).

### 2.3 Phylogenetic reconstruction

Concatenated and individual gene trees were estimated using both maximum‐likelihood (calculated with RAxML 8; Stamatakis, 2014) and Bayesian inference (MrBayes 3.2.6; Ronquist & Huelsenbeck, 2003) methods, after identifying the best‐fitting evolutionary models and partition schemes (i.e., with the lowest Bayesian information criterion score) with PartitionFinder v2.1.1 (Lanfear, Calcott, Ho, & Guindon, 2012). ML phylogenies were reconstructed with an initial search for the best tree, and additional 10,000 bootstrap replicates were run to assess nodal support of the former. Bayesian phylogenies were inferred with two independent replicate runs, each consisting of four chains running for 10 M generations. The MCMC trace was sampled every 1,000 generations, and an initial 25% was discarded as burn‐in. Convergence was assessed with Tracer v1.6 (Rambaut, Suchard, & Drummond, 2014), after which the SumTrees.py script from the DendroPy library (Sukumaran & Holder, 2010) extracted the maximum clade credibility tree (MCCT) and its nodal support as posterior probabilities.

### 2.4 Divergence time estimates and calibration points

For the estimation of the divergence time among *A. torrentium* COI mtDNA lineages, we used the Bayesian statistical framework implemented in BEAST 2.4.0. For this purpose, two different calibration methods were employed (molecular and geological). Molecular clock rate calibrations were based on the widely used (Brower, 1994) arthropod COI mtDNA substitution rate of 2.3% pairwise sequence divergence (0.0115 substitutions per site per million years).

The geological event used as calibration point was selected considering the Cenozoic tectonic evolution of the Alpine–Carpathian–Dinaric (ACD) orogenic system. The ACD orogenic system evolved during the Paleogene–Neogene and is a consequence of several tectonic events (Schmid et al., 2008): (a) continued crustal shortening in the Alps and Dinarides produced by northward movement of the Adria plate, which produced the Dinarides exhumation and establishment of the modern mountain chain during Eocene times; (b) eastward lateral extrusion of the Alps–Carpathian–Pannonian (ALCAPA) mega‐unit; and (c) the retreat of the subducted European lithospheric slab beneath the inner Carpathians, which facilitated the advance of the ALCAPA and Tisza–Dacia mega‐units into the Carpathian embayment and controlled the development of the back‐arc‐type Pannonian Basin. Various palinspastic restorations of tectonic units in the ACD orogenic system (Balázs et al., 2016; Kováč et al., 2018; Ustaszewski et al., 2008) show that before the onset of Miocene, both the Apuseni Mountains and the Dinarides belonged to the same tectonic unit, which had been formed in Oligocene (ca. 35–30 Ma) by collision of the Adria microplate with the Tisza–Dacia microplate (e.g., Schmid et al., 2008; Kováč et al., 2016). The overall topography of the Apuseni Mountains and the Dinarides was established from that moment (Kováč et al., 2016; Mațenco, 2017; Tari & Pamić, 1998). The Apuseni Mountains are probably a relic of a much wider and continuous mountain chain that occupied the space between the Dinarides and the Carpathians at the end of Paleogene times (Kováč et al., 2018, 2016; Mațenco, 2017). Around 20 Ma as a consequence of the Carpathians and Dinaridic slab rollback, the activity of the extensional subbasins and associated detachments started near or in the Dinarides and migrated NE and E‐wards in space and time forming the Pannonian basin (Balázs et al., 2016; Matenco et al., 2016). Timing of the main extensional events of the basin is constrained by the onset of extensional magmatism and by the timing of synrift and postrift sediments. According to Balázs et al. (2016, see their figure 2), the Pannonian Basin evolution has three main phases of evolution: (a) a marine phase, which started around 17 Ma in the Great Hungarian Plain part of the Pannonian Basin (Balázs et al., 2016) and around 15.2 Ma in the Southern Pannonian Basin (Sant, Palcu, Mandic, & Krijgsman, 2017); (b) a lacustrine phase starting around 11.6 Ma (ter Borgh et al., 2013); and (c) a deltaic to fluviatile phase starting around 5 Ma. As a consequence of the extensional phase in the Pannonian Basin and concomitant folding and thrusting in the Carpathians, the Apuseni Mountains, as part of the Tisza–Dacia mega‐unit, were gradually translated and clockwise rotated toward east, reaching the present‐day position around 11 Ma (Balázs et al., 2016; Roşu et al., 2004; Ustaszewski et al., 2008). During this movement, the Apuseni Mountains were above sea level and only their southern part was affected by volcanism and development of small basins (Mațenco, 2017). Between 16 and 5 Ma, the terrestrial connection between the Apuseni Mountains and the Dinarides was blocked by the existence of the marine and lacustrine phase in the Pannonian Basin. The paleo‐Danube and paleo‐Tisza river systems reached the southeastern corner of the Pannonian Basin, near the junction between the South Carpathians and the Dinarides, only around 4 Ma (Magyar et al., 2013).

The tectonic separation of the Apuseni Mountains from the Dinarides enabled us to consider the calibration point for the split between APU and the closest clade of populations from Dinarides at 16 ± 1 Ma. This moment represents the start of the Tisza–Dacia microplate (including the Apuseni Mountains) final tectonic rotation (including the Apuseni Mountains) and includes the various estimates of the beginning of the marine phase in the formation of the Pannonian Basin (Balázs et al., 2016; Kováč et al., 2018; Sant et al., 2017), allowing enough time in the basin expansion to isolate the *A. torrentium* populations from the Apuseni Mountains of those from the Dinarides. This geological event has characteristics that make it an excellent calibration point candidate (Warnock, 2015): (a) It is accurately dated; and (b) the precise point in time at which the tectonic event resulted in a complete dispersal barrier is well defined.

BEAST phylogenetic analyses were run in triplicate at Florida International University's High‐Performance Computing Cluster (Panther) for 100 M generations per replicate, after which traces and sampled trees were combined and assessed for convergence using Tracer v1.6. Divergence time analyses were run using a birth–death speciation model and a relaxed molecular clock with an uncorrelated log‐normal distribution, to account for possible rate variation among lineages. Additional information on priors and operators employed can be found within the BEAST2 XML files available in Dryad (https://doi.org/10.5061/dryad.t72v91c). Twenty‐five percent of the sampled trees were subsequently discarded as burn‐in, and nodal support as posterior probabilities was annotated on the MCCT of each BEAST tree (TreeAnnotator; Drummond & Rambaut, 2007). The geoscale.phylo function of the R package “strap” (Bell & Lloyd, 2015) was then employed to plot these phylogenies and associated divergence time estimates on a geological timescale for subsequent inferences.

### 2.5 Spatial analysis

Spatial approaches may reveal processes describing the connectivity or spreading patterns of populations (Getzin et al., 2015; Kubisch, Degen, Hovestadt, & Poethke, 2013; Pagel & Schurr, 2012). The location of each sequenced sample was georeferenced, and the geographical distance between pairs was extracted with the Network Analyst functionality of ArcGIS (version 9.3; Environmental Systems Resource Institute, Redlands, California). Pairwise genetic distances were estimated from the COI dataset using the Kimura 2‐parameter (K2P) model as implemented in MEGA 7 (Kumar, Stecher, & Tamura, 2016; da Silva et al., 2011). To avoid overparameterization and its associated biases, the K2P model was chosen for its simplicity and suitability for intraspecific estimations of pairwise genetic distances at the population level (Nei & Kumar, 2005). Associations among genetic and geographical distances between populations of each haplogroup having sufficient data for this computation (CSE: *n* = 120; GK: *n* = 15; SB: *n* = 21; ŽPB: *n* = 15) were assessed by Mantel tests based on Spearman correlation. These were performed using the package *ecodist* (Goslee & Urban, 2007) in R, version 3.3.2 (R Core Team, 2016).

## 3 RESULTS

### 3.1 Sequence data

A total of 59 new sequences were recovered for COI and 47 for 16S. The final alignments included additional 159 and 89 individual sequences, for COI and 16S, respectively, which were sourced from GenBank to include other geographically proximal *A. torrentium* populations and outgroups. A final concatenated alignment was then built using those individual specimens for which both loci were available (99 in total, 1,059 base pairs in length). All new sequence data from this project were curated, annotated, and deposited via NCBI in the GenBank database (Supporting information Table S1).

### 3.2 Haplotype networks

**Figure 2:**
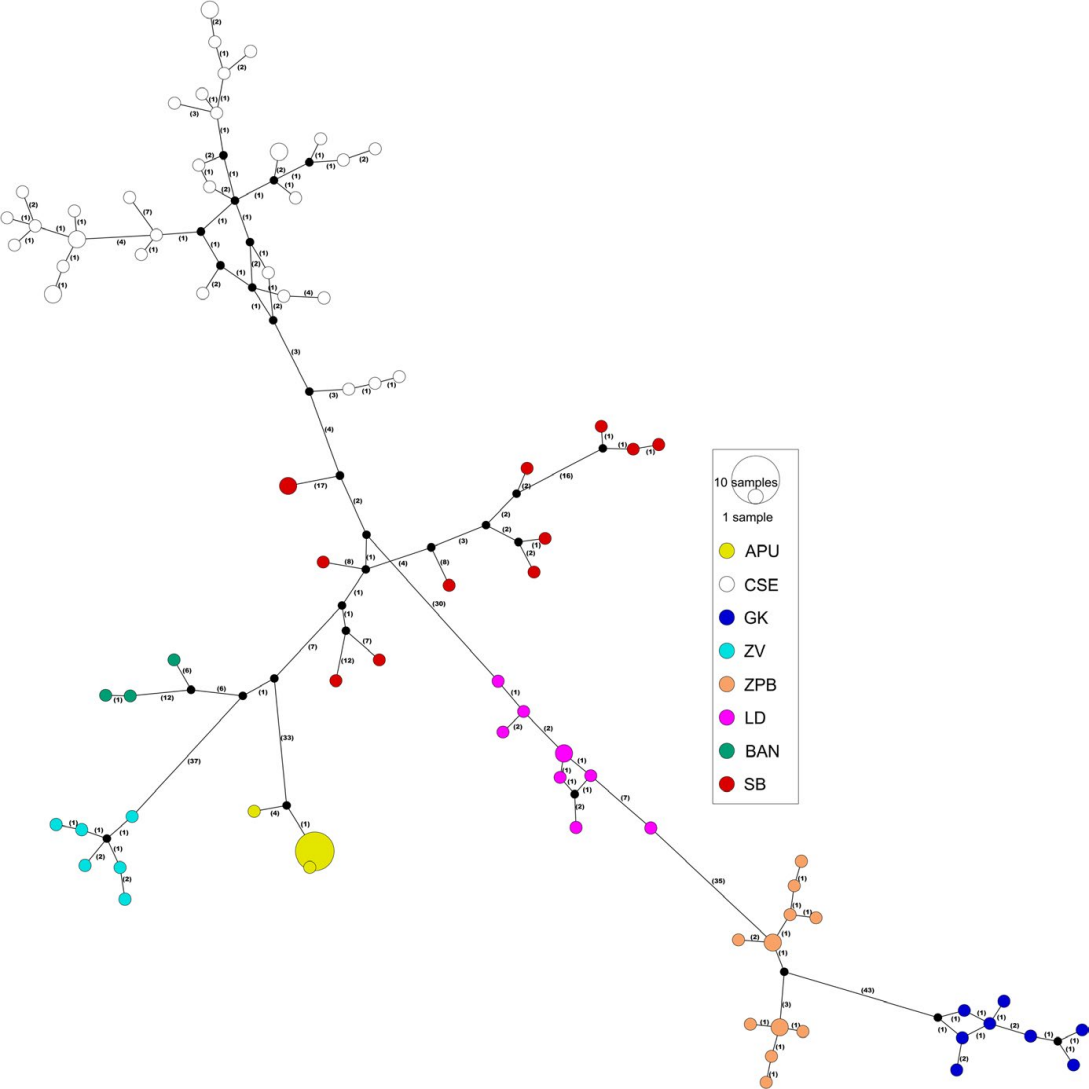
Haplotype networks of *Austropotamobius torrentium* mitochondrial loci (16S and COI). Node diameter and annotation denote sample sizes. Colors represent haplogroup, as illustrated in the legend of Figure 1

A total of 83 unique concatenated mtDNA (COI + 16S) haplotypes were recovered (Figure 2). A new haplogroup from the Apuseni Mountains, Romania (APU), was revealed in the combined mtDNA haplotype network analysis (Figure 2) and in the individual 16S and COI haplotype networks (Supporting information Figure S1). This haplogroup, which consisted of two distinct haplotypes (sequences obtained from 21 individuals), was distantly related to the adjacent haplogroups by >33 mutational steps. The remaining 81 haplotypes belonged to the populations CSE, GK, ZV, ŽPB, LD, BAN, and SB (see Figure 1 for abbreviations). The results indicate deep evolutionary divergences within *A. torrentium,* supported by a large number of mutational steps between groups. Still, the number of mutational steps between neighboring haplotypes within groups was small (1–7) with the exception of the SB and BAN groups.

### 3.3 Phylogenetic analysis

**Figure 3:**
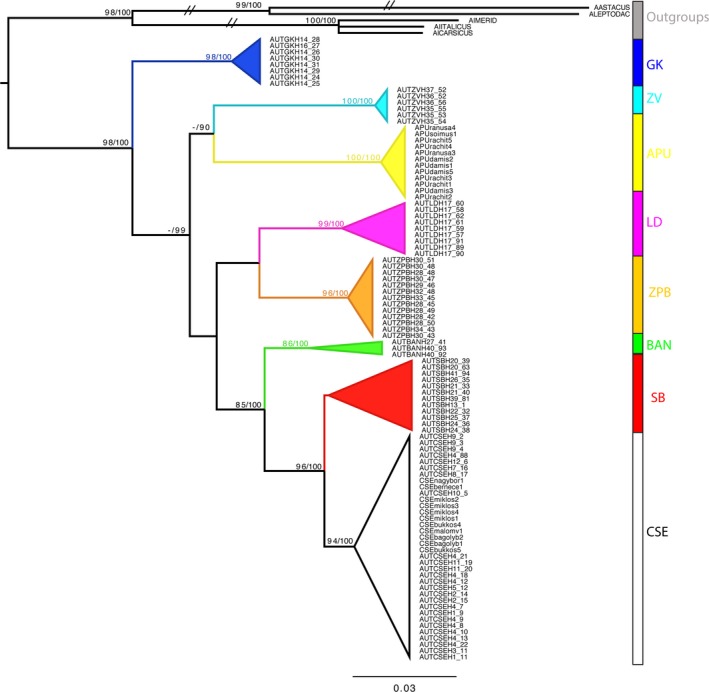
Maximum‐Likelihood phylogram estimated using concatenated mitochondrial gene data (16S and COI) showing the genetic structuring among *A. torrentium* populations. Nodes are annotated with bootstrap support values and with the posterior probabilities obtained from the equivalent Bayesian analysis

Following the computation of log‐likelihood scores for all models of evolution available in PartitionFinder, the models of best‐fit were selected based on BIC scores. This resulted in an optimal partitioning scheme consisting of a single partition for the 16S mitochondrial gene (HKY + G model) and codon‐based partitioning for COI (HKY + G, K80 + I, and F81 + I models for each position, respectively). The combined mtDNA phylogenetic analysis recovered eight monophyletic clades, seven of which were statistically supported (Figure 3). The seven supported clades included the populations GK, ZV, APU, LD, ŽPB, BAN, and CSE. The SB population was recovered to be monophyletic, albeit with low support. GK was the earliest branching lineage and sister to the remaining populations. The APU population was recovered as a sister clade to the ZV population. A strong relationship was recovered between the SB and CSE populations and with BAN as sister to SB‐CSE clade. Some of the deeper relationships among populations were poorly supported, likely due to conflict in gene trees and lack of resolution of the loci. individual gene trees (COI and 16S, Supporting information Figure S2) differed slightly, with 16S forming a polytomy among GK, APU, and ŽPB, and recovering a sister relationship between LD and ZV, however, with low support. The COI gene tree had an identical topology to that of the combined mtDNA phylogeny, according to the higher number of variable positions of this gene.

### 3.4 Divergence time estimates

**Figure 4:**
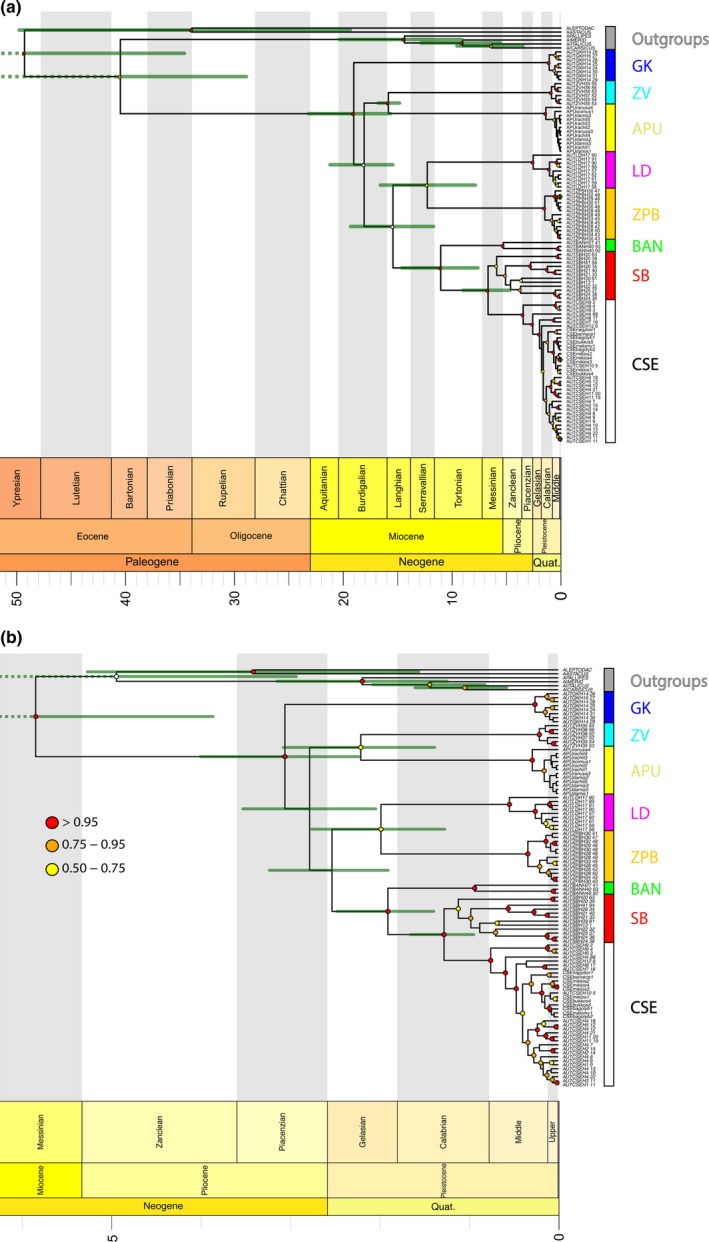
Divergence times (*x*‐axis in millions of years) of *Austropotamobius torrentium* as estimated with the proposed geological calibration (a) and arthropod substitution rate (b) in BEAST. Nodes with support values over 0.5 are annotated as indicated in the legend. Node bars depict the 95% highest posterior density (HPD) interval of the divergence between the labeled haplogroups

Divergence times were estimated using two different calibration points: (a) a geological event that represented the tectonic displacement of the Tisza–Dacia microplate and the beginning of the marine phase in the formation of the Pannonian Basin around 16 Ma and (b) a molecular clock based on a commonly used arthropod COI mtDNA substitution rate (2.3% pairwise sequence divergence or 0.0115 substitutions per site per million years; Brower, 1994). The geological time tree (Figure 4a) recovered the most recent common ancestor (MRCA) of *A. torrentium* to be ~20 Ma (HPD 24–16 Ma), with the GK lineage originating at this time. The remaining populations originated during the Burdigalian around 18 Ma (HPD 15.8–21.5). The APU population split from the ZV population around 16 Ma (HPD 15–17), represented by the calibration point of the Tisza–Dacia mega‐unit disruption of the Dinarides. The LD‐ ŽPB‐BAN‐SB‐CSE populations evolved at the same time (16 Ma, HPD 12.8–19.3). The LD‐ŽPB divergence occurred at around 13 Ma, which is similar to the divergence between BAN and SB‐CSE populations. The youngest population split is the one of CSE, which had the most recent common ancestor appearing in the Zanclean around 4 Ma (HPD 2.9–6).

The arthropod molecular clock analysis shows drastically younger ages, when compared to the geological divergence analysis (Figure 4b). Using 2.3% as substitution rate for COI (equivalent to 0.0115 substitutions per site per million years), the MRCA of *A. torrentium* only emerged ~3 Ma (HPD 2.2–4), along with the GK population. All other populations originated in the Piacenzian, soon thereafter ~2.7 Ma (HPD 2–3.5). The APU population would have diverged from the ZV population around 2.2 Ma (HPD 1.3–3), LD split from ŽPD ~1.9 Ma (HPD 1.2–2.7) and BAN split from SB‐CSE at the same time (1.9 Ma, HPD 1.4–2.5). The MRCA of the youngest population, CSE, originated in the Middle Pleistocene around 0.75 Ma (HPD 0.5–1). It should be noted that our estimates based on the substitution rate from Brower (1994) differ greatly from previous estimates recovered in Trontelj et al. (2005) or Klobučar et al. (2013). This difference was likely due to deficient quality control and trimming procedures of the raw sequencing data from previous studies, which resulted in an inflated number of observed substitutions. An overestimation of base pair substitutions would certainly lead to erroneous phylogenetic inferences as well as to distorted divergence time estimates. In the interest of reproducibility and transparency, we have included all BEAST XML files (including multiple sequence alignments) and nexus files used in this study as supplementary materials in the Dryad repository (https://doi.org/10.5061/dryad.t72v91c).

### 3.5 Spatial analysis

The spatial distribution of haplotypes shows distinct areas of spread for each haplogroup with virtually no overlap (Figure 1). The Mantel tests revealed significant positive monotonic associations between genetic and geographical distances within each haplogroup (Table 1). The relationship was strongest for the ŽPB haplotypes (*r* = 0.837) and weakest for the CSE haplotypes (*r* = 0.137). However, visual inspection of the scatter plots of genetic distance versus geographical distance reveals that, in the case of CSE haplotypes, there are numerous pairs of populations separated by large geographical distances for which the genetic distance was zero (data not shown). Since this could be indicative of two distinct spreading events, a supplementary Mantel test was performed, after having removed populations with zero genetic distance from others in the haplogroup. The association between geographical and genetic distances for the remaining 28 populations was much stronger (*r* = 0.357).

**Table 1:** Mantel *r* coefficients describing the monotonic association between genetic and geographical distances for each haplogroup, and corresponding one‐tailed *p*‐values (null hypothesis: *r* ≤ 0), computed by using 10,000 permutations

Haplogroup	*R*	*p *value
GK	0.545	0.0116
ŽPB	0.837	0.0003
SB	0.338	0.0133
CSE	0.137	0.0135
CSE > 0	0.357	0.0007

Supporting information Figure S3 highlights the pairs of CSE populations with large geographical distances, but zero genetic distances. The greatest haplotype diversity within CSE haplotypes can be found in the southern and eastern parts of the range. Nine populations located in Slovenia, northwestern Croatia, northeastern Italy, and the south of Austria all belong to haplotype 9, which also appears to be the source of a single population in the Czech Republic. Apart from these populations, central Europe also shares haplotypes 5 and 65.

## 4 DISCUSSION

### 4.1 The geological context of *A. torrentium* evolution

**Figure 5:**
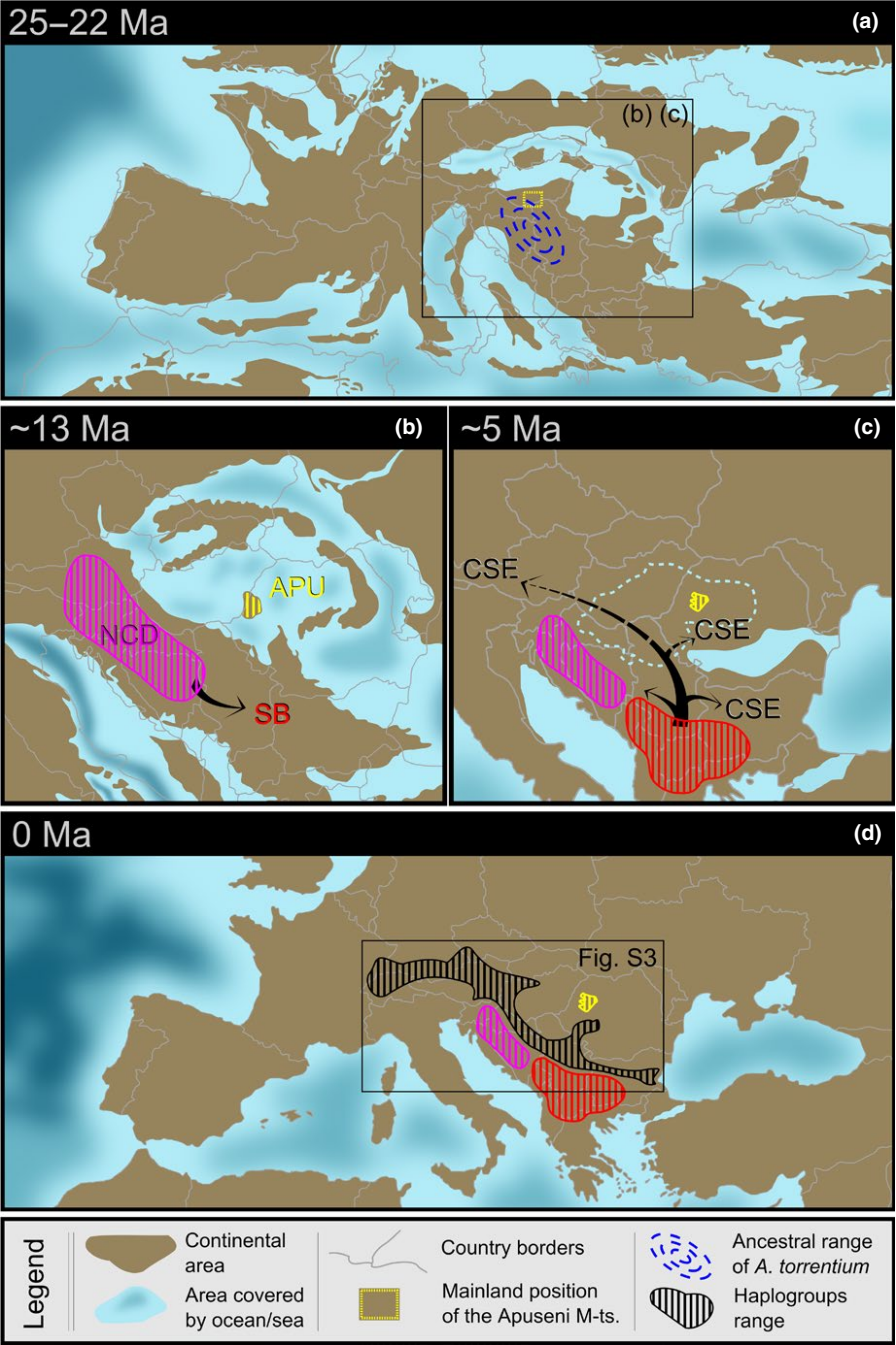
Paleogeographical reconstruction and *Austropotamobius torrentium* lineage evolution from the estimated species origin (a), followed by NCD and APU lineages separation during the tectonic displacement of the Tisza–Dacia mega‐unit (b), SB to CSE divergence and spread limited by the Pannonian Lake (c), to the current distribution after the formation of the paleo‐Danube (d). Maps used with permission of Colorado Plateau Geosystems Inc. (https://deeptimemaps.com), slightly modified after Kováč et al. (2016) and Magyar et al. (2013)

Our molecular investigations reveal that the populations of *A. torrentium* in the Apuseni Mountains are endemic, highly divergent, and related to endemic NCD populations. We suggest that this molecular relationship, which defines the geographical distance, can be explained by the paleogeography of the Dinarides and the Tisza–Dacia mega‐unit during Oligocene and Lower Miocene times. Between 35 and 16 Ma, both the Dinarides and the Tisza–Dacia mega‐unit, including the Apuseni Mountains, were part of the same continental region (Figure 5a), which was probably populated by the ancestors of *A. torrentium*. The separation of the Dinarides from the Tisza–Dacia mega‐unit (including the Apuseni Mountains), which begun at ~16 Ma, and the start of the Pannonian Basin marine phase, resulted in the isolation of the populations in the Apuseni Mountains. The Apuseni Mountains, as a part of the Tisza–Dacia mega‐unit, continued their northeasterly movement and rotation until 11 Ma, by which time they had reached their present location (Figure 5a‐c). The northern part of the Apuseni Mountains remained above sea level during these movements, providing a refuge for the ancestral population of *A. torrentium*. For ~11 million years (m.y.), the Apuseni Mountains formed a large island that was cut off from the Dinarides by the Paratethys Sea initially and then later by the Pannonian Lake. The freshwater system of the Apuseni Mountains came back in contact with other freshwater systems in the area ~5 Ma (see expanded justification in the next section below).

In a more general context for crayfish evolution, our findings suggest that the diversification of species of *Austropotamobius* took place earlier than assumed, at ~42 Ma (mean value; HPD 54–32 Ma), and the split between *Astacus *and *Austropotamobius* occurred ~48.8 Ma (HPD 62.4–37.5), which is in concordance with the timeframe estimated by Bracken‐Grissom et al. (2014) based on a Bayesian Posterior Branching Process (*BPBP*) model (Wilkinson et al., 2011). We propose that the divergence between species of *Austropotamobius* was triggered by the northward movement of the Adriatic plate, due to the Africa plate drift during the Eocene (Schmid et al., 2008). It is worth noting that other freshwater species in the Apuseni Mountains are also more closely related to species found in the Dinarides than to those found in the Carpathians (Copilaş‐Ciocianu & Petrusek, 2015; Kontschán, 2015; Meleg, Zakšek, Fišer, Kelemen, & Moldovan, 2013; Ribera et al., 2010).

### 4.2 The evolution of endemic versus widespread *A. torrentium* haplogroups

The clustering of old, endemic haplotypes of *A. torrentium* within a small geographical area of the north‐central Dinarides indicates a close and long‐lasting isolation in that area. The spatial distribution of the analyzed NCD haplogroups (GK, ŽPB) showed the highest correlation between geographical and molecular distances (Table 1). Dinarides exhumation and establishment of the modern mountain chain took place during Eocene times (Schmid et al., 2008) as a result of the convergence of the Adria plate with the European mainland started during the latest Cretaceous (Ustaszewski et al., 2009). We propose that the NCD lineages evolved in response to the continuous deformation of the Dinarides during the Miocene (Tari & Pamić, 1998; Vlahović, Tiśljar, Velić, & Matičec, 2005), which led both relief and population fragmentation. Therefore, the NCD *A. torrentium* populations are likely to have evolved under geographical isolation, due to the formation of the mountain range of Alps to the northwest, the Adriatic Sea in the south and southwest, and the Paratethys in the north (Figure 5) and subsequently the Pannonian Lake, for a period of about 11 m.y. (Balázs et al., 2016). This time frame coincides with the split and diversification of the inhabiting *A. torrentium* NCD lineages (Figure 4a). The APU haplogroup shows the same clustered pattern, since the corresponding populations are found inhabiting a very small geographical area, even though their spatial correlations could not be computed because of the low number of populations and the limited amount of available data. It is quite plausible that the endemism of the *A. torrentium* populations in the Apuseni Mountains resulted from the isolation of that particular geographical unit in an island shape (Figure 5b) that lasted for about 11 m.y. (Balázs et al., 2016). This hypothesis is supported by other studies on freshwater species that have explained their endemism as being due to insularity that resulted from being cut off by saline water (Costedoat & Gilles, 2009; Neubauer, Harzhauser, Georgopoulou, Kroh, & Mandic, 2015).

The SB haplogroup, currently distributed within the Balkan Mountains, evolved more recently and has a relaxed spatial distribution that is well reflected in molecular distances (Table 1). These populations split from the southeastern Dinarides (at ~16 Ma) and expanded progressively toward the east and south (Figure 5b,c), favored by the improved freshwater hydrological connectivity in the southeastern part of the Dinarides. The starting of this expansion can also be triggered by the disappearance of the Dinarides Lake System around 16 Ma (Kováč et al., 2018; Mandic et al., 2012; Sant et al., 2017). Next, the start of the deltaic phase and the development of the paleo‐Danube system within the Pannonian Basin (at ~5 Ma) provided suitable conditions for the spread of and already spread *A. torrentium* SB lineage, thus forming the CSE lineage (Figure 5c) which steadily colonized a large area extending from the Balkans to central Europe (Figure 5d). The east–west direction of colonization is supported by the spatial visualization of haplotypes shown in Supporting information Figure S3. These two scenarios explain the conservative spreading behavior of NCD and APU populations in contrast with the two SB and CSE lineages expressing more expansiveness during history.

The current distribution of the most recent populations within the CSE haplogroup seems to have been influenced by two different drivers from the other *A. torrentium* haplogroups. Our spatial analyses revealed a weak correlation between genetic and geographical distances for those populations. The spatial correlation improved significantly when the population pairs sharing the same haplotypes were removed (Table 1). Supporting information Figure S3 shows that the geographical distance is influenced by a small number of paired haplotypes that made a far more significant contribution to the migration activity than the other haplotypes. We propose that these populations may be a result of postglacial colonization. Some of these populations are also likely to have been influenced by translocations, as has been suggested by a number of other authors (Fratini et al., 2005; Petrusek et al., 2017; Trontelj et al., 2005). Further insights into population genetics and additional information on ecological contexts (such as possible temporary freshwater drainage connections, migration pathways, repopulation programs, and even fluvial transport routes) may shed further light on this matter.

### 4.3 Revisiting molecular clock calibrations

Current thinking is that the Dinarides orogeny was responsible for triggering the separation of *A. pallipes* from *A. torrentium* (see Trontelj et al., 2005), and this event has therefore been used to calibrate relaxed molecular clocks with mean ages varying between 16 and 12.5 Ma (depending on the author; e.g., Klobučar et al., 2013; Jelić et al., 2016). The phylogenetic reconstruction of *A. torrentium*, including the APU lineage, clearly shows two major colonization events in the Pannonian Basin as opposed to the single major spread of the CSE lineage presented in previous studies. Attempts to fit these two events within the time frame suggested by previous authors failed to explain some important aspects. The above time frame constrained the divergence of the APU lineage to ~2.2 Ma (HPD 1.3–3), but since the Pannonian Basin was at that time in the paleo‐Danube phase, the colonization of such a highly specific restricted area as the Apuseni Mountains cannot be reasonably explained. Present‐day Tisza River and its tributaries, which could have been used by *A. torrentium* to reach the northern part of the Apuseni Mountains, were formed after 5 Ma (Magyar et al., 2013). Moreover, even if it was accepted that there was a large‐scale expansion from the Dinarides that crossed the large Pannonian Plane, it would still be difficult to explain why only individuals of one NCD‐related haplogroup (namely the ZV) migrated if ecological conditions were roughly the same for all of them. Our argument for a different time frame is further supported by the results of Copilaş‐Ciocianu and Petrusek (2015) who calculated a date of ~19 Ma (HPD 13.6–26.2) for the divergence of a freshwater amphipod population in the Apuseni Mountains, which is in turn consistent with the geological evidence of the Tisza–Dacia mega‐unit separation.

**Figure 6:**
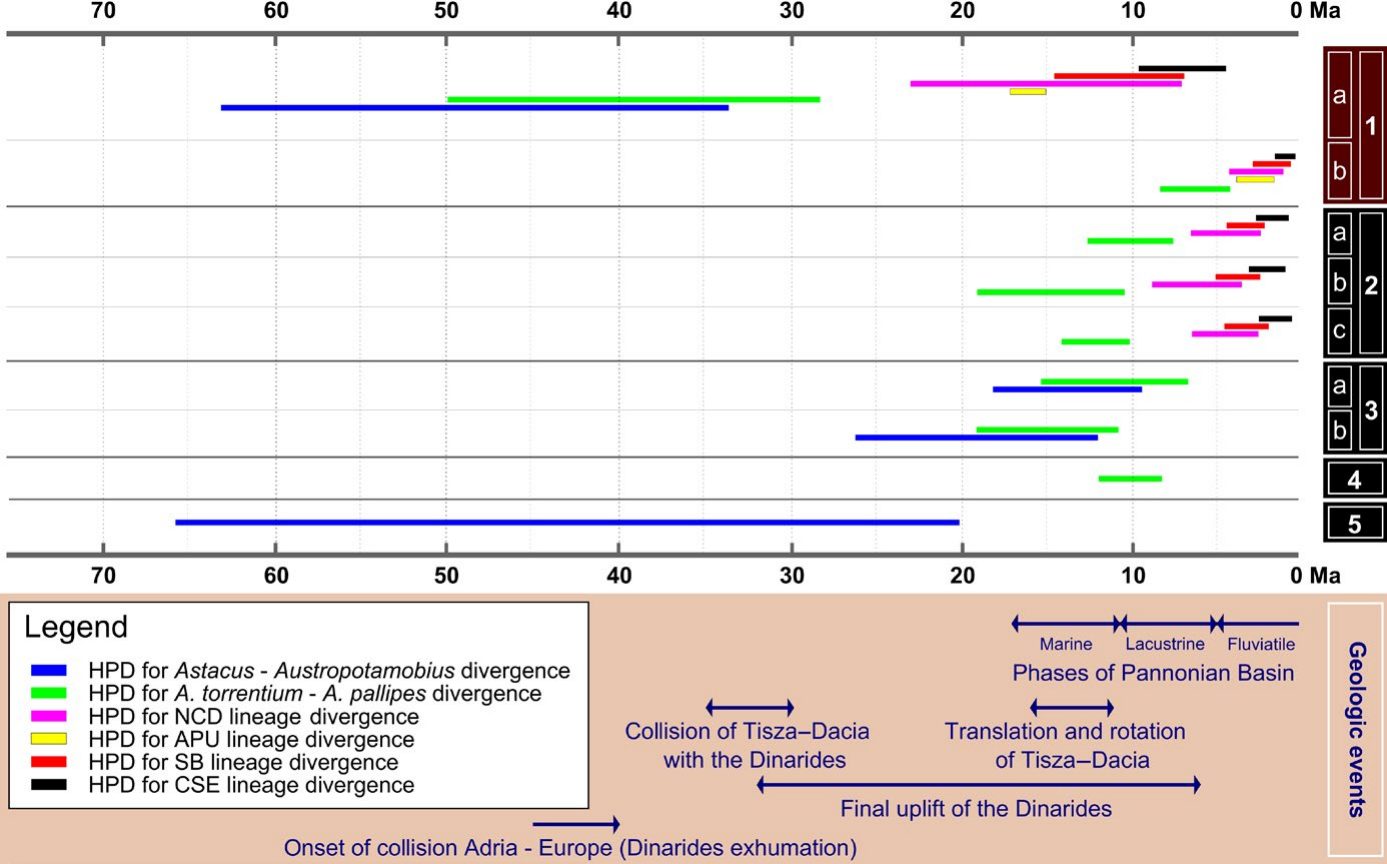
Comparison of HPD divergence time chronogram estimates for the native European astacids obtained (1) in this study using (a) tectonic calibration, and (b) arthropod substitution rate, with those from other previous studies (2) by Klobučar et al. (2013) using (a) arthropod substitution rate, (b) crustacean substitution rate, and (c) geological calibration; (3) by Jelić et al. (2016) using (a) arthropod substitution rate, and (b) geological calibration; (4) by Trontelj et al. (2005) using crustacean substitution rate; and (5) by Bracken‐Grissom et al. (2014) using a *BPBP* model. For details of the methods, the reader is referred to the above‐mentioned papers and references therein. For information on geological events in the figure, see Schmid et al. (2008), Balázs et al. (2016), and Kováč et al. (2016)

A comparison between the HPDs of the molecular clock estimated in this study and that estimated in other studies by Trontelj et al. (2005), Klobučar et al. (2013), Jelić et al. (2016), and Bracken‐Grissom et al. (2014) is available in Figure 6. Our estimates based on tectonic calibration do not overlap with any of the HPD results based on substitution rates or calibration based on the Dinarides orogeny, as provided by Trontelj et al. (2005), Klobučar et al. (2013), and Jelić et al. (2016). An overlap occurred with the HPD of the two European astacid genera divergences estimated by Bracken‐Grissom et al. (2014) based on a *BPBP* fossil calibration model. Using a different approach based on a *28‐fossil* age calibration method, Bracken‐Grissom et al. (2014) obtained an HPD of 128.5–110 Ma, which is similar to the 110 Ma obtained by Owen, Bracken‐Grissom, Stern, and Crandall (2015) on the basis of molecular data. Papers by Trontelj et al. (2005), Klobučar et al. (2013), and Jelić et al. (2016) reported younger HPD estimates based on arthropod or crustacean substitution rates, or using the Dinarides orogeny calibration fixed at 16 or 12.5 Ma. In light of our results, the younger molecular time estimates for the Dinarides appear likely to have been based on a calibration point inadequate for the taxa of interest. In view of the long and complex uplift history of the Dinarides during the Oligocene and Miocene periods (Figure 6), the authors using this calibration event did not present any convincing geological evidence that only those episodes of uplift around the mean values of 16 Ma or 12.5 Ma resulted in an effective barrier against dispersal. Moreover, the substitution rates used for calibration based on Brower (1994), Schubart, Diesel, and Hedges (1998), Lefébure, Douady, Gouy, and Gibert (2006), and others, may be inappropriate in this case, as they are based on much more recent geological events and on distantly related taxa. Recent studies involving *A. torrentium *(i.e., Klobučar et al., 2013; Jelić et al., 2016) that employ molecular clock calibrations have used COI substitution rates estimated by Brower (1994). However, these rates were originally estimated from several different regions of the mitochondrial genome, obtained through a variety of methods, of seven arthropod taxa. Six of these taxa consisted of insects, and only one was a crustacean (*Alpheus *spp. shrimp; Brower, 1994). As such, the authors themselves not only suggest being cautious of the data's large compounded error, but even designate the rates’ generalized applicability for Arthropoda as preliminary (Brower, 1994). Moreover, substitution rates inferred from recent divergences (<1–2 Ma) have been found to be remarkably different than those at deeper levels (Ho, Phillips, Cooper, & Drummond, 2005). This transition between short‐term and long‐term substitution rates is measurable, explained by an exponential decay curve, and unlikely to be an artifact (Ho et al., 2005). Divergence time estimations inferred with substitution rates from different timescales are often invalid unless corrected with the aforementioned curve (Ho et al., 2005). To the best of our knowledge, no previous study of *A. torrentium* that used Brower, (1994) substitution rates has performed this correction in their analyses. Finally, there is recent evidence for substitution rate variation among populations inhabiting different latitudes (Schär et al., 2017). These substitution rate variations, which were also observed by Trontelj et al. (2005) in *Austropotamobius*, further emphasize the need for proper and careful consideration of evolutionary timescales and substitution rates when conducting molecular dating analyses.

## 5 CONCLUSIONS

Phylogenetic reconstruction of a species based on molecular data requires full geographical coverage. Previous species phylogenies of *Austropotamobius torrentium* were incomplete, because the northeastern part of the species distribution had not been included. This study fills that data gap, which turns out to be one of the most important pieces of the puzzle. Molecular analyses reveal that the populations sampled from the Apuseni Mountains are very different from nearby populations, but surprisingly related to populations found about 600 km away in the Dinarides. We explain the endemism of these populations (i.e., the APU haplogroup) in the Apuseni Mountains through the well‐established plate tectonic history of this area: the Tisza–Dacia mega‐unit (which includes the Apuseni Mountains), which broke away from a larger plate that included the Dinarides and traveled toward the northeast during the Miocene. The mega‐unit carried with it part of the *A. torrentium* population and kept it isolated for ~11 m.y. as the plate was surrounded by saline water (first by the Paratethys and later on by the Pannonian Basin).

Alternative scenarios based on traditional molecular clock calibrations failed to explain the endemism of the APU haplogroup. We propose that the calibrations that were used misinterpreted the timing of the separation between *A. pallipes* and *A. torrentium *within the context of the Dinarides orogeny. The timing of this split was fixed subjectively at mean values of either 16 or 12.5 Ma, but the uplift of the Dinarides was a much longer process that started in the Eocene and continued during the Miocene and into the Quaternary.

All these cumulative evidences might constrain the crayfish evolution in the area to a much earlier time frame. The large genetic separation of the APU haplogroup lineage may have interesting taxonomic implications. We therefore raise the question of whether this haplogroup might be eligible for consideration as a new species, or as a subspecies of *A. torrentium*, as was previously proposed for the endemic haplogroups in the Dinarides by Klobučar et al. (2013).

## CONFLICT OF INTEREST

None declared.

## AUTHOR CONTRIBUTIONS

LP conceived the idea and collected crayfish field samples, partially supplemented by AW and BG; I‐DP under the supervision of AS performed molecular analyses; JLP‐M and HBG performed molecular data analyses and processing; CZ performed spatial analyses; CP and LD carried out of geological and geographical context; LP, CP, LD, HBG, and CDS wrote the first version of the manuscript; and all authors contributed and approved the final version.

## Data Availability

All sequences used this study are accessible via GenBank, and accession numbers can be found in Supporting Information.
