# Supplementary figures and images for: A journey on plate tectonics sheds light on European crayfish phylogeography
Source: Ecol Evol. 2019 Jan 19;9(4):1957–71. doi: 10.1002/ece3.4888 (PMC6392496; doi:10.1002/ece3.4888)

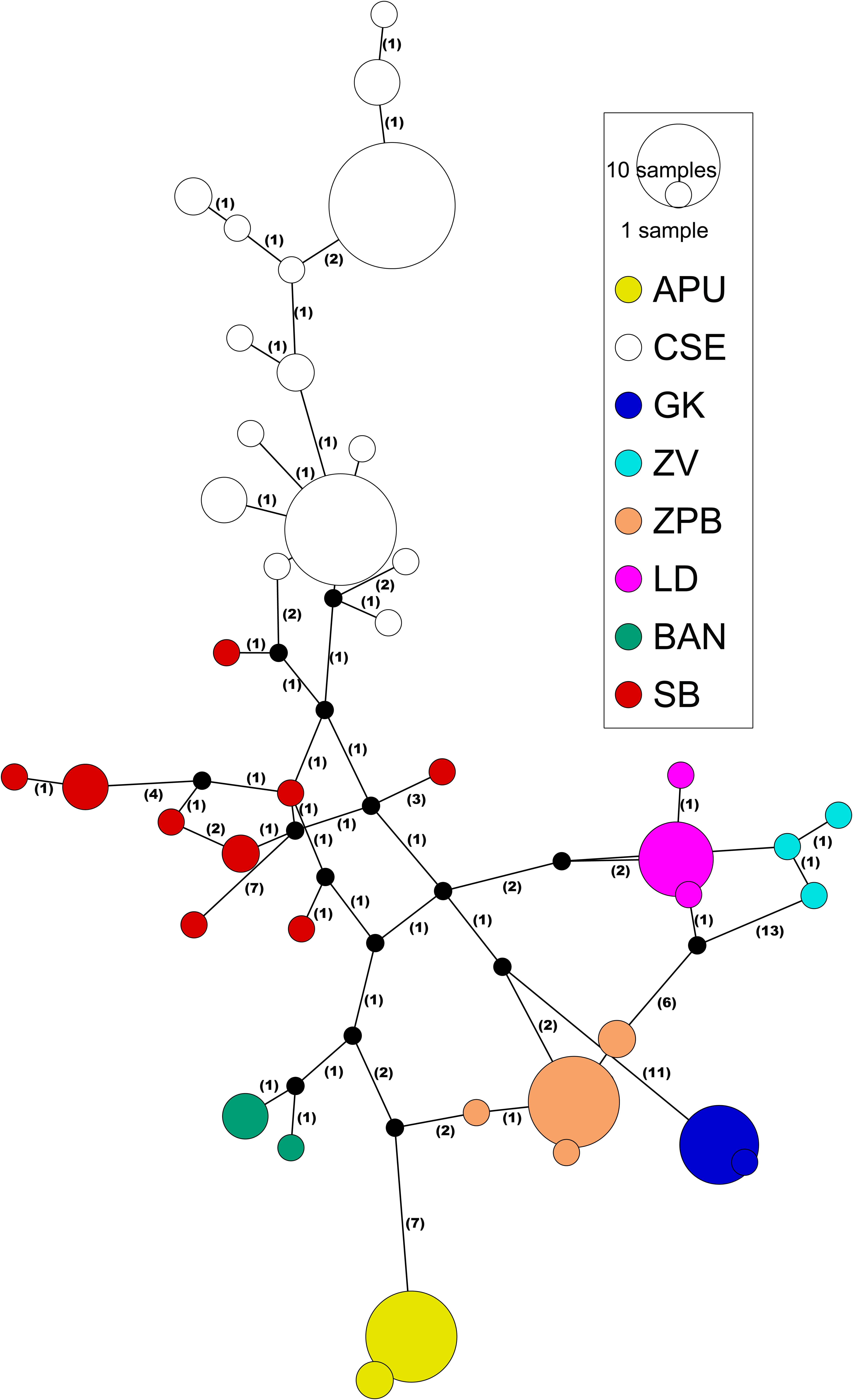

Supplement: Supplementary file 1 [file ECE3-9-1957-s001.tif]

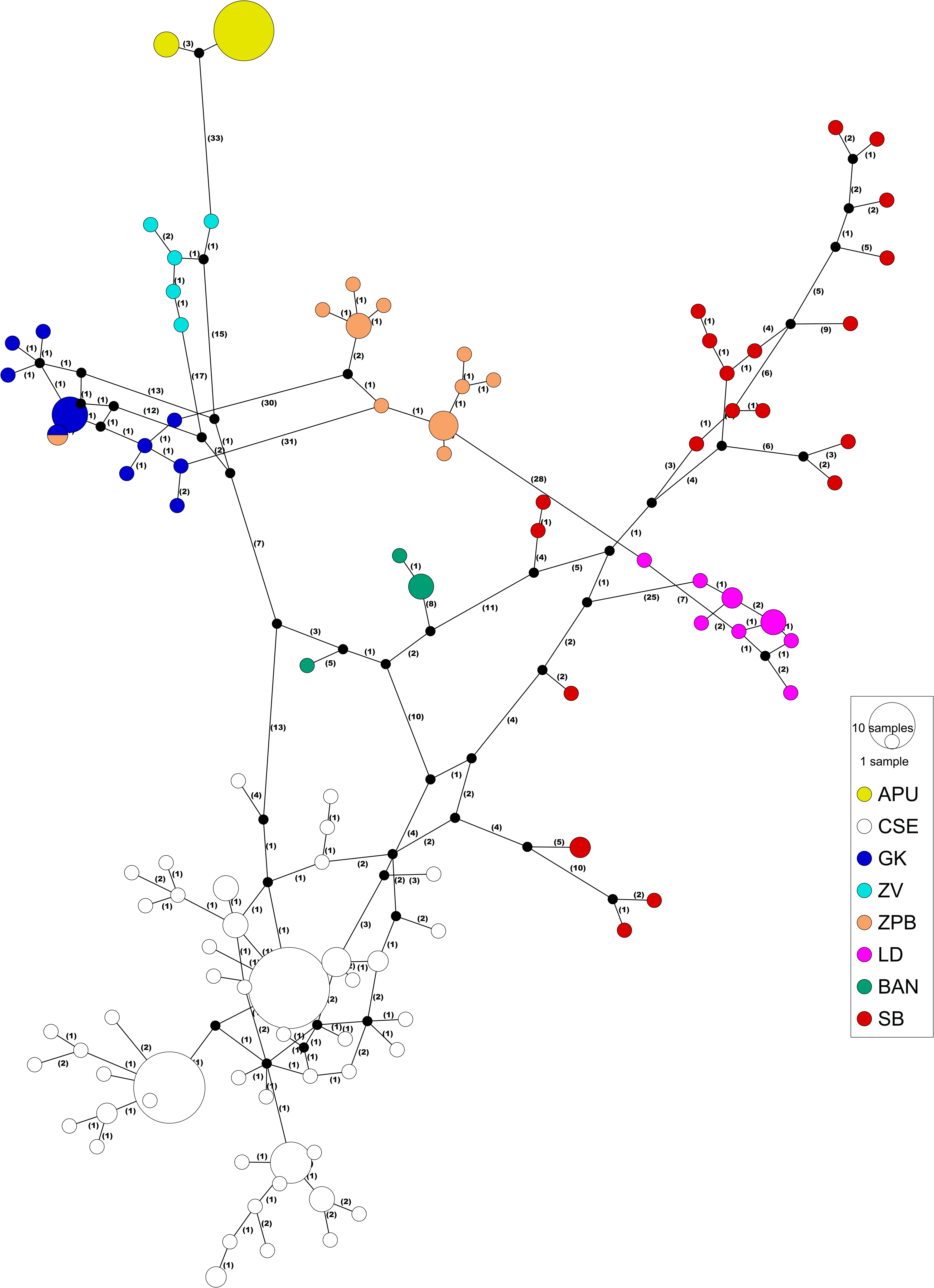

Supplement: Supplementary file 2 [file ECE3-9-1957-s002.tif]

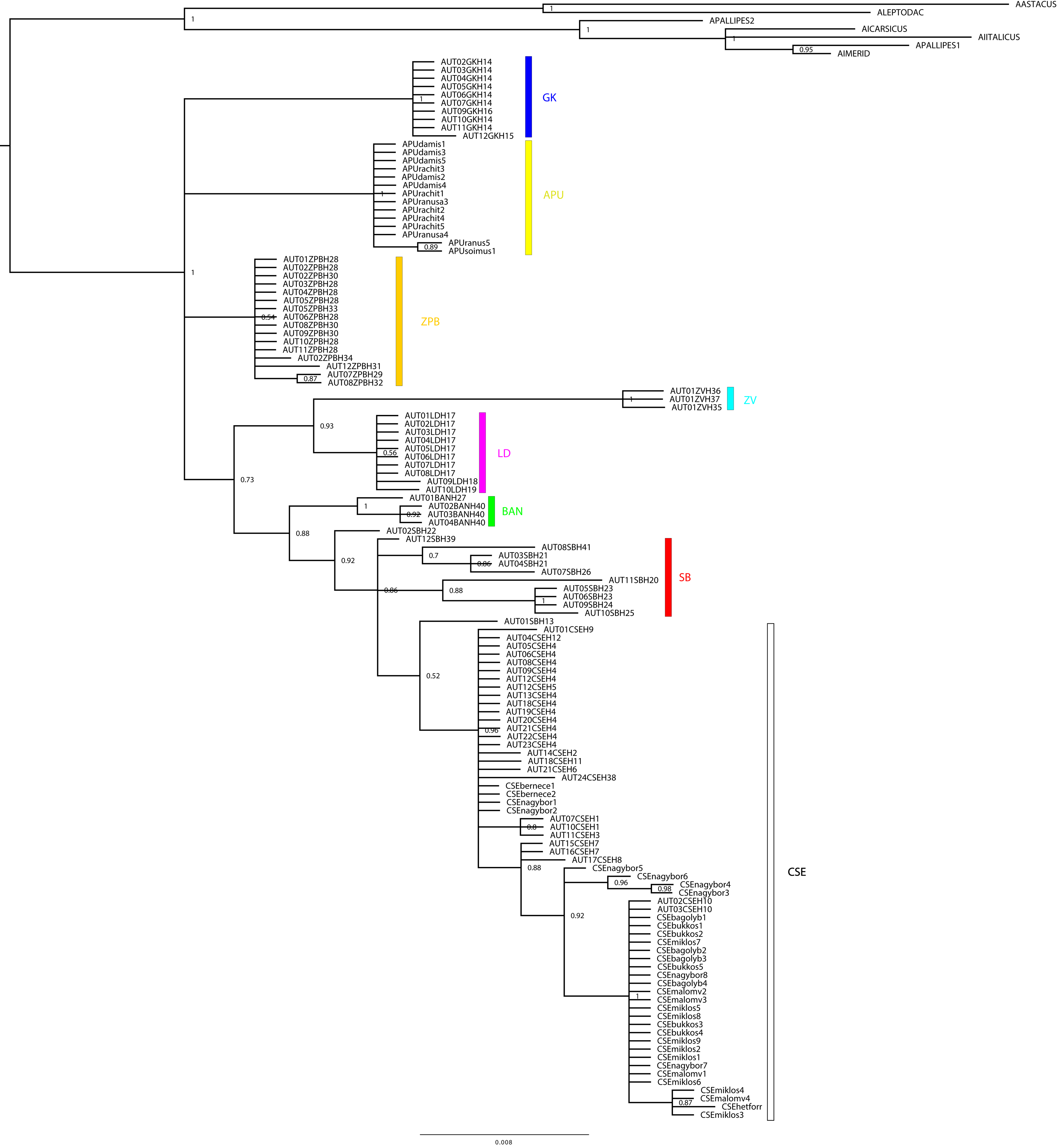

Supplement: Supplementary file 3 [file ECE3-9-1957-s003.tif]

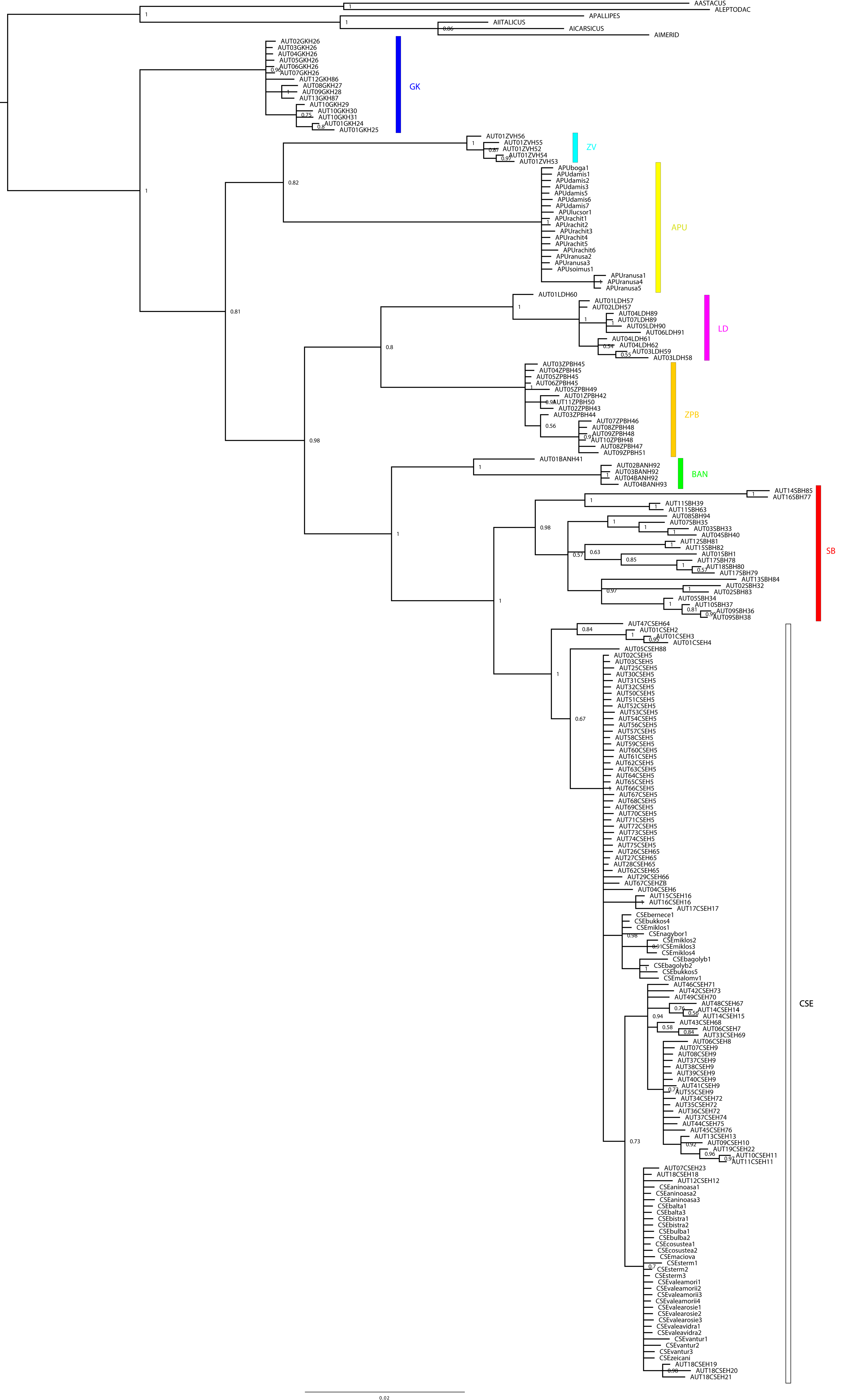

Supplement: Supplementary file 4 [file ECE3-9-1957-s004.tif]

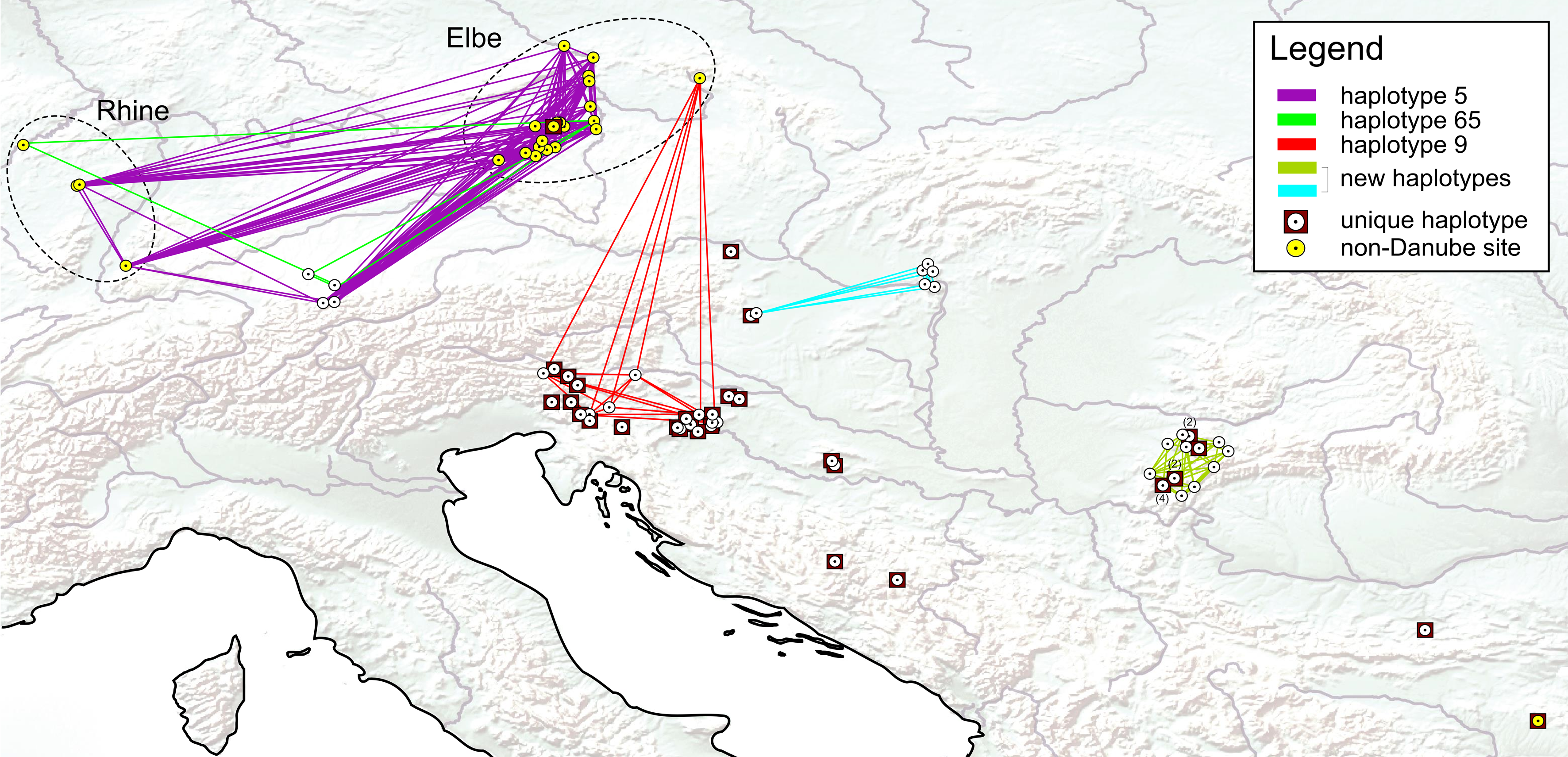

Supplement: Supplementary file 5 [file ECE3-9-1957-s005.tif]
